# Diagnostic research in immune checkpoint inhibitor-related pneumonitis: a bibliometric analysis of research evolution, diagnostic focuses, and future priorities

**DOI:** 10.3389/fonc.2026.1885789

**Published:** 2026-07-13

**Authors:** Luwei Han, Wei Zhang, Qi Zhang, Wenhao Shen, Ping Yang, Bing Fang, Yuanxin Meng, Yi Liu, Ziyan Ren, Ruiyang Fei, Xinran Zhang, Jiaxing Li, Kaijin Lu, Gaohua Han

**Affiliations:** 1 Dalian Medical University, Dalian, China; 2 Department of Oncology, The Affiliated Taizhou People’s Hospital of Nanjing Medical University, Taizhou, China; 3 Department of Thoracic Surgery, The Affiliated Taizhou People’s Hospital of Nanjing Medical University, Taizhou, China

**Keywords:** biomarkers, bronchoalveolar lavage, diagnostic assessment, differential diagnosis, imaging features, immune checkpoint inhibitor-related pneumonitis, risk prediction

## Abstract

**Background:**

Immune checkpoint inhibitor-related pneumonitis (CIP) is an important pulmonary toxicity in cancer immunotherapy. Its diagnostic assessment is difficult because symptoms and imaging overlap with infection, radiation pneumonitis, tumor progression, pre-existing interstitial lung disease, and other drug-induced lung injuries. Although publications on CIP have increased in recent years, research on diagnostic assessment remains insufficiently described.

**Methods:**

Publications on CIP and diagnostic assessment were retrieved from the Web of Science Core Collection (WoSCC) and PubMed. After screening, 628 WoSCC records were included as the main dataset for bibliometric analysis, and 74 PubMed records were used for supplementary clinical-topic assessment. Microsoft Excel, VOSviewer, CiteSpace, Bibliometrix, and SCImago Graphica were used to examine publication trends, country and institutional contributions, author collaboration, journal distribution, keyword co-occurrence, clustering, burst terms, and PubMed-based diagnostic topic classification.

**Results:**

CIP research was sparse before 2015, increased after 2015, and accelerated after 2019, with the highest annual output observed in 2023. China published the highest number of papers, while the United States has the greatest citation impact. Institutional and author analysis identified active research groups mainly from China, the United States and Japan. Research is mainly published in oncology, immunology, thoracic medicine and respiratory journals. Keyword analysis shows a shift from melanoma, T cells and immune checkpoint inhibition towards clinically focused themes, including diagnosis, differential diagnosis, imaging features, bronchoalveolar lavage (BAL), biomarkers, risk factors and nomogram-based prediction. A supplementary PubMed analysis similarly highlights imaging features, BAL, bronchoscopy or pathological assessment, and risk prediction as the primary clinical diagnostic themes.

**Conclusion:**

Research into CIP has evolved from the recognition of immune-related pulmonary toxicity to diagnostic assessment, differential diagnosis and personalised risk assessment. Current diagnostic studies reflect the clinical challenge of distinguishing CIP from other pulmonary complications in patients receiving immunotherapy. Future research should focus on developing and validating standardised, multicenter diagnostic strategies linked to prognosis. Current research trends suggest that imaging findings, bronchoalveolar lavage fluid or histopathological assessment, biomarkers, baseline lung function, and predictive models may form key components of such strategies; however, their clinical utility requires validation through prospective studies.

## 1 Introduction

Immune checkpoint inhibitors (ICIs) have become an integral part of modern cancer treatment. By targeting immune inhibitory pathways such as programmed cell death protein 1, programmed death ligand 1 and cytotoxic T-lymphocyte-associated antigen 4 (1), these drugs not only restore anti-tumour immune activity but also improve treatment outcomes for a wide range of malignancies (2–4). However, with the widespread clinical use of ICIs, immune-related adverse events have become increasingly common in routine cancer care. Among these, Immune checkpoint inhibitor-related pneumonitis (CIP) warrants particular attention. This is because lung injury can rapidly deteriorate, necessitating the interruption of anti-tumour therapy; in severe cases, it can lead to respiratory failure or even death (5–7).

Diagnostic assessment of CIP is not straightforward. Most patients typically present with cough, dyspnoea, chest tightness or low-grade fever, and some patients initially present with imaging abnormalities alone. However, these manifestations can easily be confused with infection, tumour progression, radiation pneumonitis, pulmonary oedema, pre-existing interstitial lung disease or other drug-induced lung injury (3, 8, 9). Chest CT is central to clinical assessment, but its radiological findings are not specific indicators. For instance, CIP patients often present with ground-glass opacities, changes resembling organized pneumonitis, interstitial changes, or diffuse inflammatory shadows; however, a variety of chest lesions may also exhibit similar radiological features (7, 8, 10–12). Furthermore, with the advancement of multidisciplinary teams, an increasing number of patients are receiving combination therapies such as chemotherapy combined with immunotherapy, radiotherapy combined with immunotherapy, and targeted therapy combined with immunotherapy, which has further complicated the differentiation between CIP and other treatment-related pulmonary toxicities (10, 13–16).

Consequently, the diagnostic assessment of CIP is increasingly viewed as a multidimensional clinical challenge rather than a straightforward diagnosis. The patient’s medical history, the onset of symptoms (17), CT findings (18), tests for markers of infection (19), bronchoscopy or bronchoalveolar lavage (BAL) (20) where necessary, pathological information (21), and circulating biomarkers (22) may all provide evidence to support the diagnostic assessment. In recent years, many researchers have begun to focus on serum biomarkers (22–24), inflammatory cytokines, radiomics, PET/CT results (25) and risk (26) models, among other areas. This indicates that the field is shifting from simple case identification towards diagnostic assessment, differential diagnosis and risk stratification. Nevertheless, the existing evidence remains scattered across disciplines such as oncology, respiratory medicine, radiology, pathology and immunology, making it difficult to grasp the overall direction of research based on individual studies alone.

Although numerous reviews and clinical guidelines have summarized the diagnosis and management of CIP, they typically focus on specific clinical evidence or expert recommendations. These publications do not sufficiently highlight the evolution of the field over time, which countries and institutions have driven its development, which journals have published key evidence, or which diagnostic topics are gradually gaining attention. Through bibliometric analysis, we can quantitatively examine publication output, collaboration patterns, source distribution, keyword associations, topic clustering and temporal bursts, thereby helping to address these questions. For topics concerning clinical practice and mechanistic research related to the diagnostic assessment of CIP, this approach can serve as a useful complement to traditional review methods (27).

Consequently, this study focuses on the literature relating to the diagnosis of CIP, with particular emphasis on the diagnostic assessment and differential diagnosis of CIP. Using the Web of Science Core Collection (WoSCC) as the primary data source, this study analyzed trends in the growth of the literature, contributions by country and institution, author collaboration, journal distribution, and the clustering and evolution of keywords. In addition, we conducted a separate search of PubMed to provide a clinically oriented comparison of the diagnostic topic. By analyzing these two datasets, we describe the development and trends in international research on the diagnostic assessment of CIP in recent years.

## 2 Materials and methods

### 2.1 Data sources and search strategy

This study utilised the WoSCC as the primary data source for bibliometric analysis, as its comprehensive citation records and bibliographic fields offer significant advantages for investigating collaborative relationships between countries and institutions, the distribution of sources, citation intensity, and keyword clustering. In addition, a separate search was conducted in PubMed; after excluding studies already included in WoSCC, the results were treated as studies relevant to clinical diagnosis.

**Figure 1:**
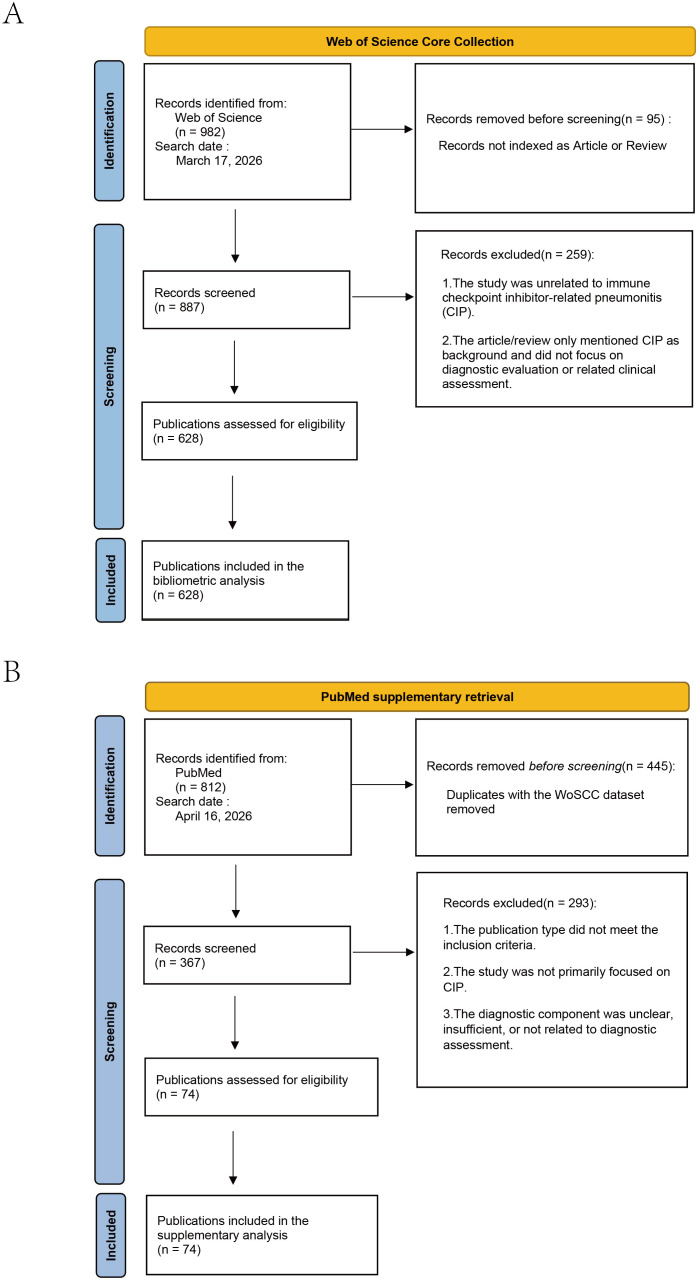
Flowchart of literature retrieval and study selection. **(A)** Screening process of the Web of Science Core Collection (WoSCC) dataset used for the main bibliometric analysis. **(B)** Supplementary retrieval and screening process of the PubMed dataset used for supplementary thematic analysis.

The WoSCC database was searched on 17 March 2026 and used as the primary dataset for the bibliometric analysis. To provide supplementary clinical subject information, the PubMed database was searched on 16 April 2026. As the PubMed dataset was used solely for supplementary subject analysis rather than for independent bibliometric analysis, the difference in search dates is not expected to have a substantial impact on the overall interpretation of research trends. In the WoSCC database, a combined search was performed using terms such as immune checkpoint inhibitors, checkpoint inhibitor-associated pneumonitis, immune-related pneumonitis and diagnostic assessment (**Supplementary Material**). The initial search yielded a total of 982 records. Records were restricted to those indexed as Article or Review in WoSCC. Because some records carried multiple document-type labels, records indexed as Article or Review were retained even when secondary labels (e.g., Proceedings Paper or Early Access) were also present. After this restriction, 887 records proceeded to title and abstract screening. Following manual screening, 259 articles were excluded because they had no direct association with CIP, mentioned CIP only as background information, or did not focus on diagnostic assessment or related clinical assessment. Ultimately, 628 articles were retained as the primary dataset for the bibliometric analysis (**Figure 1A**).

We employed a similar search strategy to conduct a supplementary search in the PubMed database. A total of 812 records were initially identified. After excluding 445 duplicate records that overlapped with the WoSCC dataset, the remaining 367 records proceeded to the screening stage. Of these, 293 were excluded due to insufficient relevance to the diagnostic assessment of immune-mediated pneumonitis. Ultimately, a total of 74 PubMed articles were included in the supplementary thematic analysis (**Figure 1B**).

Due to differences between the WoSCC and PubMed databases in terms of citation structure, indexing rules and export formats, the records from these two sources have not been merged into a single dataset. The WoSCC dataset is used for primary bibliometric analysis, while the PubMed dataset was used as a complementary source to provide additional clinically oriented perspectives on diagnostic topics identified in the WoSCC dataset.

### 2.2 Inclusion and exclusion criteria

Included studies must meet all of the following criteria: they must be original articles or reviews; published in English; directly related to immune checkpoint inhibitor-related pneumonitis; and explicitly focus on diagnosis, diagnostic assessment, differential diagnosis, imaging features, biomarkers, bronchoscopy, pathology, risk factors or predictive models.

Records other than articles and reviews were excluded. Publications unrelated to immune checkpoint inhibitor therapy, not focused on CIP, or lacking a diagnostic component were also removed. Publications that mentioned CIP only briefly, without further diagnostic assessment, were excluded after title and abstract screening.

Two researchers independently screened the retrieved records and extracted the required information. Disagreements were resolved through discussion, and a third reviewer was consulted when consensus could not be reached. Detailed screening criteria are provided in **Supplementary Tables S1**–**S3**.

### 2.3 Data cleaning and standardization

Prior to the analysis, the data was screened for duplicate entries, spelling inconsistencies, abbreviation variants and synonymous terms. Complete records and cited references exported from WoSCC were used for the bibliometric analysis. Literature information and subject data, such as diagnoses, were extracted from PubMed for supplementary descriptive analysis.

Prior to conducting co-occurrence and clustering analyses, the keywords were cleaned. Terms with similar meanings were merged to reduce noise. For example, “inhibitor related pneumonitis”, “checkpoint inhibitor pneumonitis”, “checkpoint inhibitor-associated pneumonitis” and their associated abbreviations were all standardized to “immune-related pneumonitis”. Diagnostic terms related to imaging, radiological features, biomarkers, BAL, bronchoscopy and risk prediction were also reviewed and standardised. During this process, custom alias files in CiteSpace were utilized.

### 2.4 Bibliometric analysis and visualization

Microsoft Excel 2022 was used for data organization and descriptive statistics, including annual publication trends in the WoSCC.

VOSviewer version 1.6.20 was used to build collaboration networks among institutions and authors. The total counting method was used. To make the maps easy to read, the minimum publication threshold was set at 6 for authors and 5 for institutions. In the network maps, node size showed publication output, link thickness showed collaboration strength, and node color showed collaboration clusters.

CiteSpace version 6.4.R1 was used to perform keyword co-occurrence analysis, keyword clustering, timeline visualization and detection of emerging keywords. The time span was set from 2008 to 2026, with each time segment covering one year. Keywords were selected as the node type. Node selection was performed using the g-index, where k = 25. The network structure was clarified by applying the Pathfinder algorithm, pruning the segmented networks, and pruning the merged network. The final keyword network comprised 266 nodes and 763 links, with a density of 0.0216. Burst detection was performed using γ = 0.6 and a minimum burst duration of at least one year.

Bibliometrix 5.1.0, running on R version 4.5.0, was used to analyze national-level output, publications from individual countries, multi-country collaborative publications, authors’ output over time, and source-related indicators. SCImago Graphica was used to visualize national-level publication output and international collaboration. Country collaboration data exported from VOSviewer was imported into SCImago Graphica, where the size of the circles represents the number of publications and the connecting lines represent collaborative relationships between countries or regions.

### 2.5 Supplementary PubMed-based clinical-topic assessment

The PubMed dataset was not used to construct a complete network, but was analyzed separately. Its purpose is to provide a clinically oriented supplement to the results of the WoSCC-based bibliometric study.

After removal of records overlapping with the WoSCC dataset, the remaining PubMed records were manually screened according to predefined inclusion and exclusion criteria (**Supplementary Table S1**). A total of 74 eligible articles were retained for supplementary analysis. These articles were subsequently categorized into six predefined diagnostic themes: clinical diagnosis and differential diagnosis; imaging characteristics; BAL/bronchoscopy/pathology; biomarkers or laboratory markers; risk factors/prediction/prognosis; and treatment or guideline-related literature. The classification criteria are provided in **Supplementary Table S2**. The thematic classification was independently performed by two investigators. Inter-rater agreement was assessed using Cohen’s kappa coefficient and demonstrated excellent agreement (κ = 0.862, P < 0.001; **Supplementary Table S4**). Disagreements were resolved through discussion and adjudication by a third reviewer when necessary. Using this classification, the clinical focus of the PubMed articles was used to supplement and compare the data included in the WoSCC dataset.

### 2.6 Reproducibility and future update strategy

To facilitate reproducibility and future updates, the complete search strategies, raw retrieved records before manual screening, final included WoSCC records, final included PubMed records, PubMed clinical-topic classification file, and CiteSpace keyword alias file were provided as **Supplementary Data S1–S7**. Future updates of this bibliometric analysis can be performed by repeating the same database-specific search strategies, applying the same predefined eligibility criteria, and using the same bibliometric and keyword-standardization parameters.

## 3 Results

### 3.1 Annual publication trends

**Figure 2:**
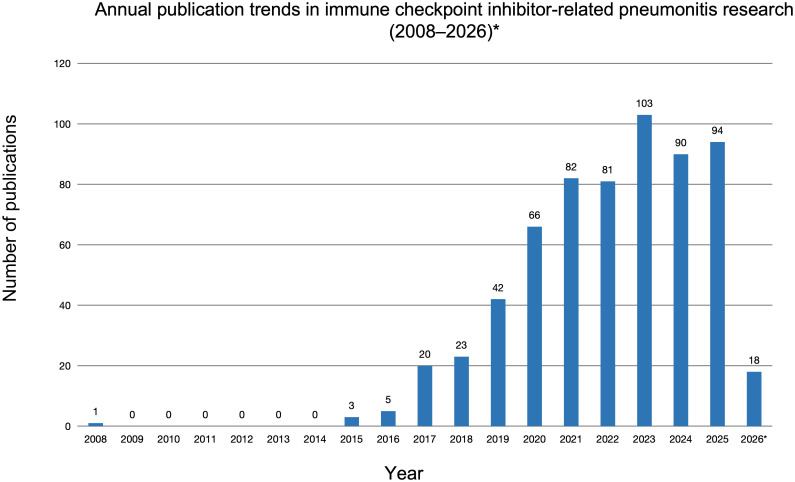
Annual publications on pneumonitis associated with immune checkpoint inhibitors from 2008 to 2026.*Data for 2026 were collected up to March 17, 2026, and do not represent the complete year. The total number of publications per year has shown an overall upward trend, with a significant increase since 2019, reaching a peak in 2023.

Early research into CIP progressed slowly. Only one eligible publication was identified in 2008, and no relevant records were found between 2009 and 2014. Publication activity began to increase from 2015 onwards, with three papers published that year; by 2018, the annual output had risen to 23 papers (**Figure 2**). This early phase is strongly correlated with the gradual adoption of ICIs in the treatment of a wider range of tumors. At the same time, immune-related lung toxicity also began to attract increasing clinical attention.

Since 2019, the upward trend has become even more pronounced. The number of relevant papers rose from 42 in 2019 to 66 in 2020, and further increased to 82 in 2021. Although there was a slight decline in 2022, with 81 papers published, annual output reached its highest level in 2023, with a total of 103 papers published. The field remained active in 2024 and 2025, with 90 and 94 papers published respectively. Consequently, international scholars have begun to recognize that CIP is not an isolated adverse event, but rather an ongoing area of research.

A total of 18 articles were identified in 2026. As the search was conducted on 17 March 2026 and did not cover the full year, this figure should be interpreted with caution. Overall, the trend in publication numbers shows a significant increase since 2019, which is consistent with the growing clinical need for improved diagnostic assessment, differential diagnosis and management of CIP.

### 3.2 Distribution of countries/regions and international collaboration

**Figure 3:**
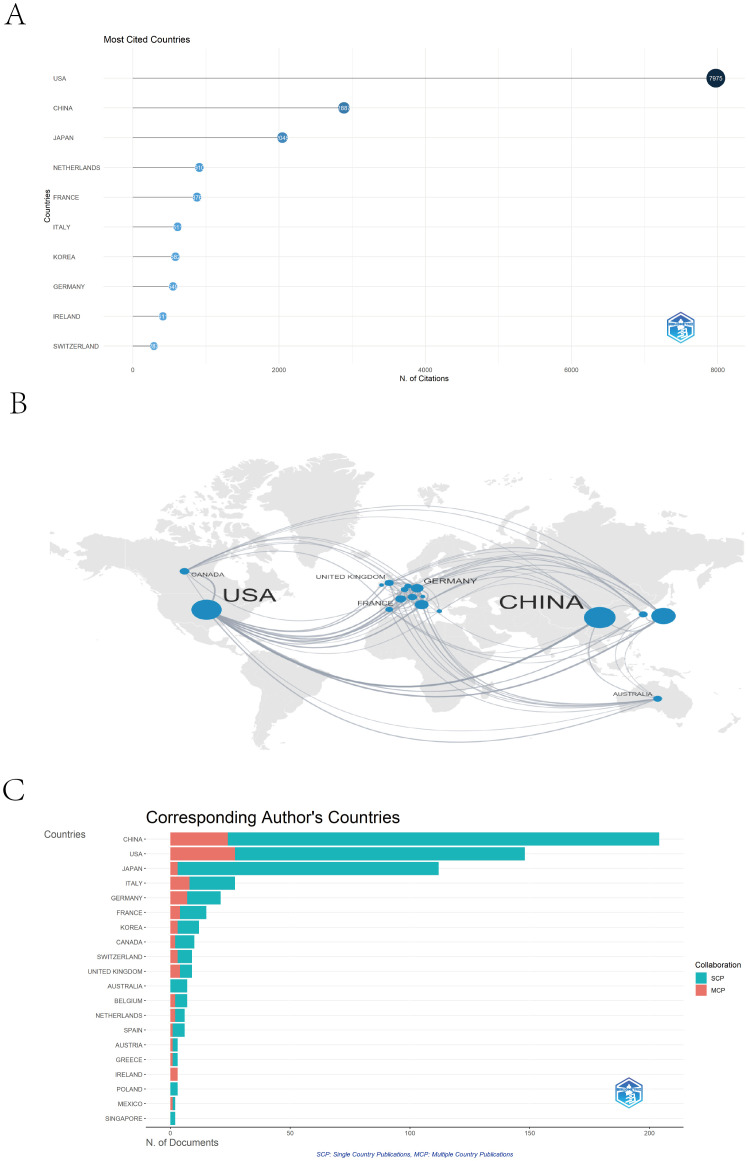
Contributions by different countries to CIP research and international collaboration. **(A)** Leading countries ranked by total number of citations. **(B)** Global collaboration network among countries. Node size represents paper output, and links indicate collaborative relationships between countries. **(C)** Distribution of papers by the country of the corresponding author, including papers published by a single country (SCP) and those resulting from multinational collaboration (MCP).

The patterns revealed by citation analysis differ from those observed in terms of paper output. The United States ranked highest with a total of 7,975 citations, followed by China with 2,887 and Japan with 2,043 (**Figure 3A**). Although China published the highest number of papers, the United States had a greater citation impact. The disparity between publication output and citation impact suggests that multi-center collaboration and high-impact clinical evidence may play an important role for future CIP research.

This diagram of international cooperation (**Figure 3B**) shows that China and the United States are two core nodes in the international network. Germany, France and the United Kingdom also occupy prominent positions within the European cooperation cluster. Consequently, the network of international cooperation can be seen to unfold among a few key contributors, rather than being evenly distributed among all participating countries.

China publishes the highest number of papers, but its multiple-country publications (MCP) stand at 11.8% (**Figure 3C**), with the majority of research constituting single-country studies. The United States has an MCP of 18.2% (**Figure 3C**), suggesting a higher level of engagement in international collaborative research. Some European countries publish fewer papers, but their proportion of collaborative research is relatively high. Among the top ten countries, the UK has the highest MCP rate at 44.4%. Meanwhile, both Germany and Switzerland have MCP rates of 33.3%. Therefore, high output does not necessarily imply stronger cross-border collaboration.

Analysis at the national level indicates that CIP research is primarily concentrated in a small number of high-output countries (**Figure 3**; **Table 1**). China tops the list, having published 204 papers, accounting for 32.5% of the WoSCC dataset. The United States accounts for 23.6% of the dataset, ranking second with 148 papers, whilst Japan accounts for 17.8% of the dataset, ranking third with 112 papers. This also demonstrates that research activity is primarily concentrated in East Asia and North America.

**Table 1:** Top 10 countries ranked by corresponding author’s publications in immune checkpoint inhibitor-related pneumonitis research.

Rank	Country	Publications	Percentage (%)	SCP	MCP	MCP ratio (%)
1	China	204	32.5	180	24	11.8
2	USA	148	23.6	121	27	18.2
3	Japan	112	17.8	109	3	2.7
4	Italy	27	4.3	19	8	29.6
5	Germany	21	3.3	14	7	33.3
6	France	15	2.4	11	4	26.7
7	Korea	12	1.9	9	3	25.0
8	Canada	10	1.6	8	2	20.0
9	Switzerland	9	1.4	6	3	33.3
10	United Kingdom	9	1.4	5	4	44.4

Although China, the United States, and Japan accounted for the largest numbers of publications, citation impact showed a somewhat different pattern (**Figure 3A**). In particular, the Netherlands and Ireland ranked among the most highly cited countries despite producing relatively few publications (**Figure 3C**), suggesting that scientific influence is not solely determined by publication quantity. These findings indicate that several countries with lower productivity have nevertheless made disproportionately influential contributions to the development of CIP diagnostic assessment.

### 3.3 Institutions and collaboration network

**Figure 4:**
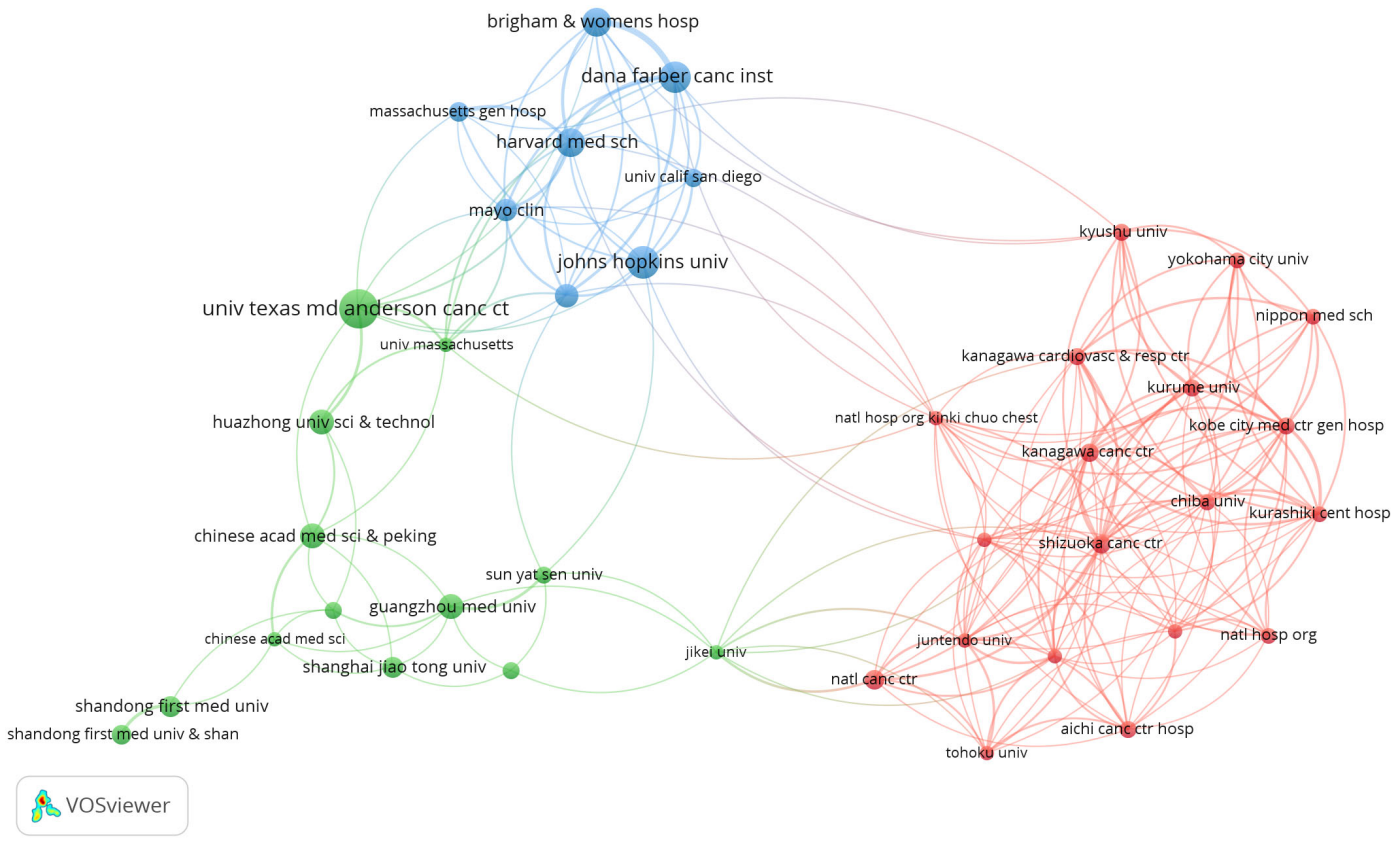
A collaborative network of institutions involved in CIP research. Node size represents research output, whilst links indicate collaborative relationships between institutions. Different colours denote different collaborative groups.

The institutional collaboration network is characterized by several stable academic groups and a marked geographical concentration. (**Figure 4**). It is evident that institutions from the United States, China and Japan occupy a prominent position, which is consistent with the distribution of research output across these countries.

The central region of the collaboration network is occupied by several major cancer centers and medical schools, including the University of Texas MD Anderson Cancer Centre, Johns Hopkins University, the Dana-Farber Cancer Institute and Shanghai Jiao Tong University. These institutions share multiple collaborative links and are primarily involved in clinical and translational research related to thoracic oncology, immunotherapy and CIP management. In addition, a Japanese collaborative cluster has been identified, involving the National Cancer Centre, the National Hospital Organization, Nippon Medical School, Chiba University and Kurume University. Compared to the extensive international connections exhibited by some US and Chinese institutions, this cluster demonstrates a stronger regional concentration. Consequently, this institutional network is composed of a limited number of influential clinical centers and regionally affiliated research teams.

### 3.4 Authors and collaboration network

**Figure 5:**
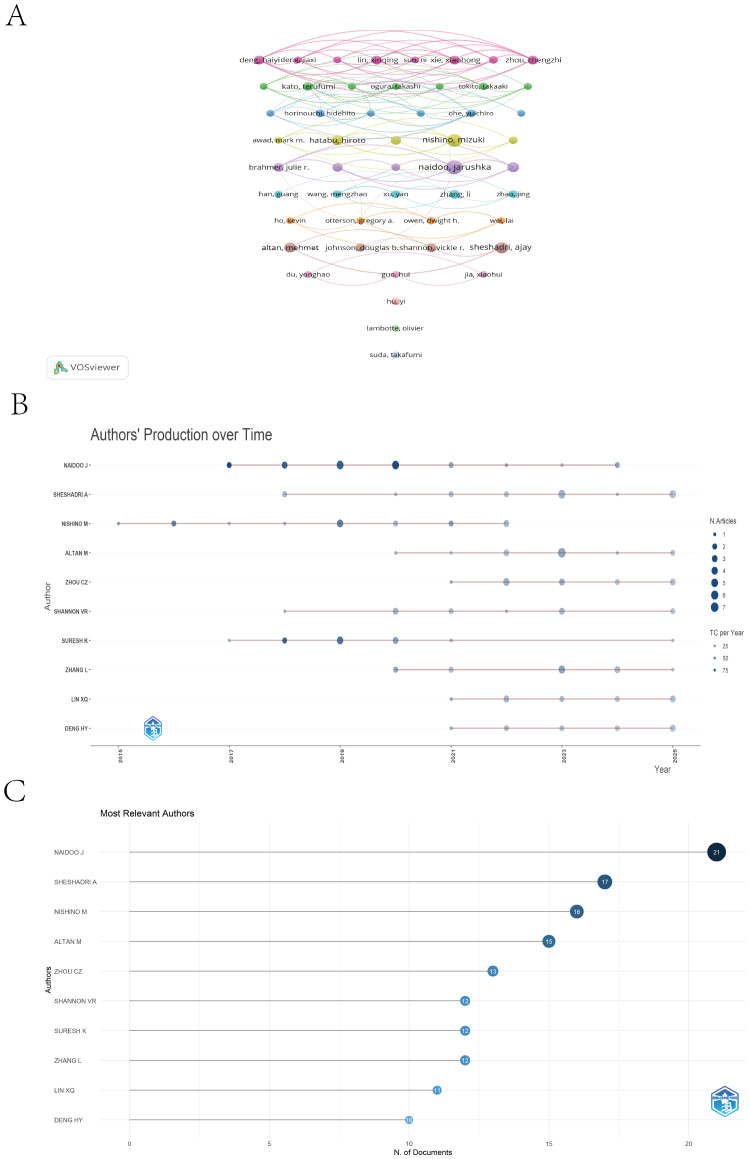
Author productivity and collaboration networks in CIP research. **(A)** Collaboration network among authors. Node size represents the number of papers, and links indicate collaborative relationships between authors. Different colours denote different author clusters. **(B)** Leading authors ranked by number of papers. **(C)** Author productivity over time. Bubble size represents the number of papers, and colour intensity reflects the total number of citations per year.

The author collaboration network reveals that there are a number of identifiable research teams in the field of CIP research, rather than being dominated by individual researchers. These teams are primarily centered around authors who have made repeated contributions to the description of the clinical features, radiological assessment and treatment management of CIP (**Figure 5A**).

Among the most productive authors, Naidoo J ranked first with 21 publications. Sheshadri A followed with 17 publications, Nishino M with 16 publications, Altan M with 15 publications, and Zhou CZ with 13 publications (**Figure 5B**). Shannon VR, Suresh K, Zhang L, Lin XQ, and Deng HY also published many studies in this field. Their work connected CIP research with thoracic oncology, immune-related adverse events, imaging interpretation, and clinical risk assessment.

Judging by the temporal distribution of the authors’ publications, Naidoo J and Nishino M focused on this field at an early stage and have maintained their influence over the long term. Meanwhile, authors such as Sheshadri A, Altan M, Zhou CZ, Lin XQ and Deng HY have gradually emerged in recent years (**Figure 5C**). Over time, the pool of authors has continued to expand, and an increasing number of researchers are turning their attention to studies on diagnosis, prediction and clinical management.

### 3.5 Journal distribution and source impact

**Figure 6:**
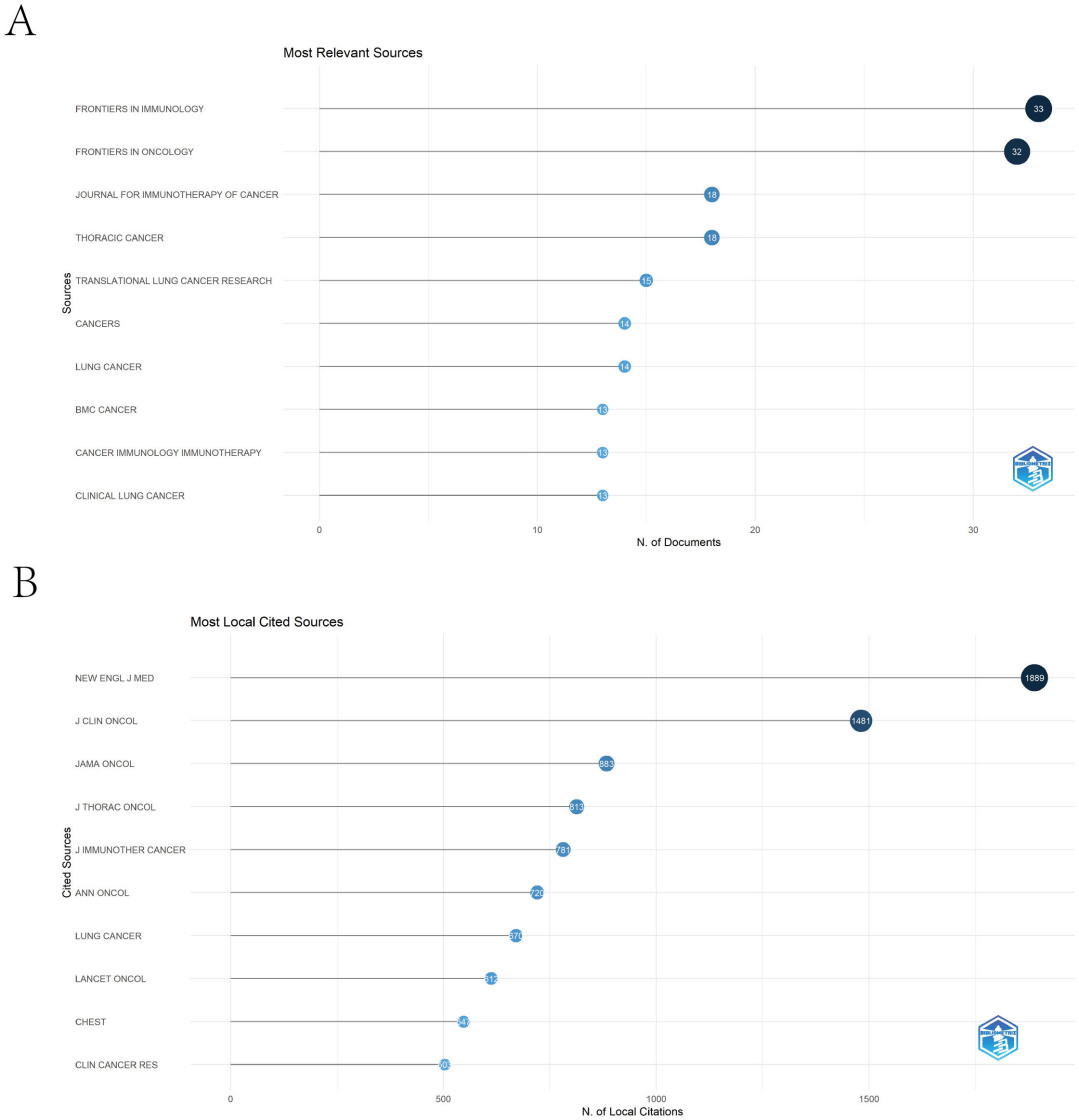
The distribution of journals and the influence of literature sources in CIP research. **(A)** Top journals ranked by number of published papers. **(B)** The most frequently cited literature sources in the dataset, ranked by the number of local citations.

Research related to CIP has been published primarily in journals specializing in immunology, oncology, thoracic malignancies and cancer treatment. *Frontiers in Immunology* was the most productive journal, with 33 publications, followed closely by *Frontiers in Oncology* with 32 publications. *Journal for Immunotherapy of Cancer* and *Thoracic Cancer* each published 18 articles. Other active journals included *Translational Lung Cancer Research*, *Cancers*, *Lung Cancer*, *BMC Cancer*, *Cancer Immunology, Immunotherapy*, and *Clinical Lung Cancer* (**Figure 6A**). This distribution indicates that CIP research lies at the intersection of tumor immunology, thoracic oncology and respiratory toxicology.

The distribution of locally cited publications differs from that of publication output. *New England Journal of Medicine* ranked first in local citations, with 1,889 citations, followed by *Journal of Clinical Oncology* with 1,481 citations and *JAMA Oncology* with 883 citations. Other frequently cited sources included *Journal of Thoracic Oncology*, *Journal for Immunotherapy of Cancer*, *Annals of Oncology*, *Lung Cancer*, *Lancet Oncology*, *CHEST*, and *Clinical Cancer Research* (**Figure 6B**). These publications form the primary clinical evidence base for cancer immunotherapy, thoracic oncology, respiratory medicine and the management of immune-related adverse events. High-impact journals underpin a sustained output of publications, while highly cited papers provide the clinical and theoretical foundation for CIP research.

### 3.6 Highly cited publications

The 10 most frequently cited papers were ranked according to their annual citation rates to identify which studies have had the greatest impact on CIP research (**Table 2**). These papers were primarily published in leading journals in the fields of oncology, immunotherapy, respiratory medicine and radiology, reflecting the clinical and multidisciplinary nature of this field of research.

**Table 2:** Top 10 highly cited publications in immune checkpoint inhibitor-related pneumonitis research ranked by annual citation rate.

Rank	First author	Year	Journal	Total citations	TC per year
1	Haanen J	2022	Ann Oncol	802	160.40
2	Naidoo J	2017	J Clin Oncol	925	92.50
3	Friedman CF	2016	JAMA Oncol	672	61.09
4	Shankar B	2020	JAMA Oncol	385	55.00
5	Chennamadhavuni A	2022	Front Immunol	262	52.40
6	Darnell EP	2020	Curr Oncol Rep	312	44.57
7	Suresh K	2018	J Thorac Oncol	358	39.78
8	Nishino M	2016	Clin Cancer Res	394	35.82
9	Suresh K	2018	CHEST	303	33.67
10	Delaunay M	2017	Eur Respir J	320	32.00

Annual citation rates were calculated using the interval between the publication year and the search date (March 17, 2026). Because 2026 represents a partial year, annual citation rates for recently published articles may be slightly underestimated.

The ESMO clinical practice guidelines published by Haanen et al. in *Annals of Oncology* in 2022 topped the list with a total of 802 citations and an annual citation rate of 160.40. This high annual citation rate reflects the practical importance of clinical guidelines in the identification, grading, treatment and follow-up of immunotherapy-related toxicities. The study by Naidoo et al., published in the *Journal of Clinical Oncology* in 2017, ranked second, with a total of 925 citations and an annual citation rate of 92.50. This study remains an important reference for the description of early clinical features of CIP.

Other widely cited studies have focused on immune-related adverse events associated with ICIs, clinical management, imaging features and pulmonary complications. Studies by Friedman et al., Shankar et al., Suresh et al., Nishino et al. and Delaunay et al. have provided important evidence regarding the clinical presentation, imaging features, incidence, severity and management of CIP. These studies have transformed CIP from an immune-related adverse event into a disease with a more clearly defined diagnostic framework.

Highly cited literature can broadly be divided into three categories: clinical guidelines on the management of immunological toxicity, studies describing the incidence and prognosis of CIP, and studies on the differential diagnosis of CIP in imaging or respiratory medicine. This classification structure reflects the complexity of CIP diagnosis, as it typically requires the joint involvement of oncologists, respiratory specialists, radiologists and other specialists.

### 3.7 Keyword co-occurrence and clustering analysis

**Figure 7:**
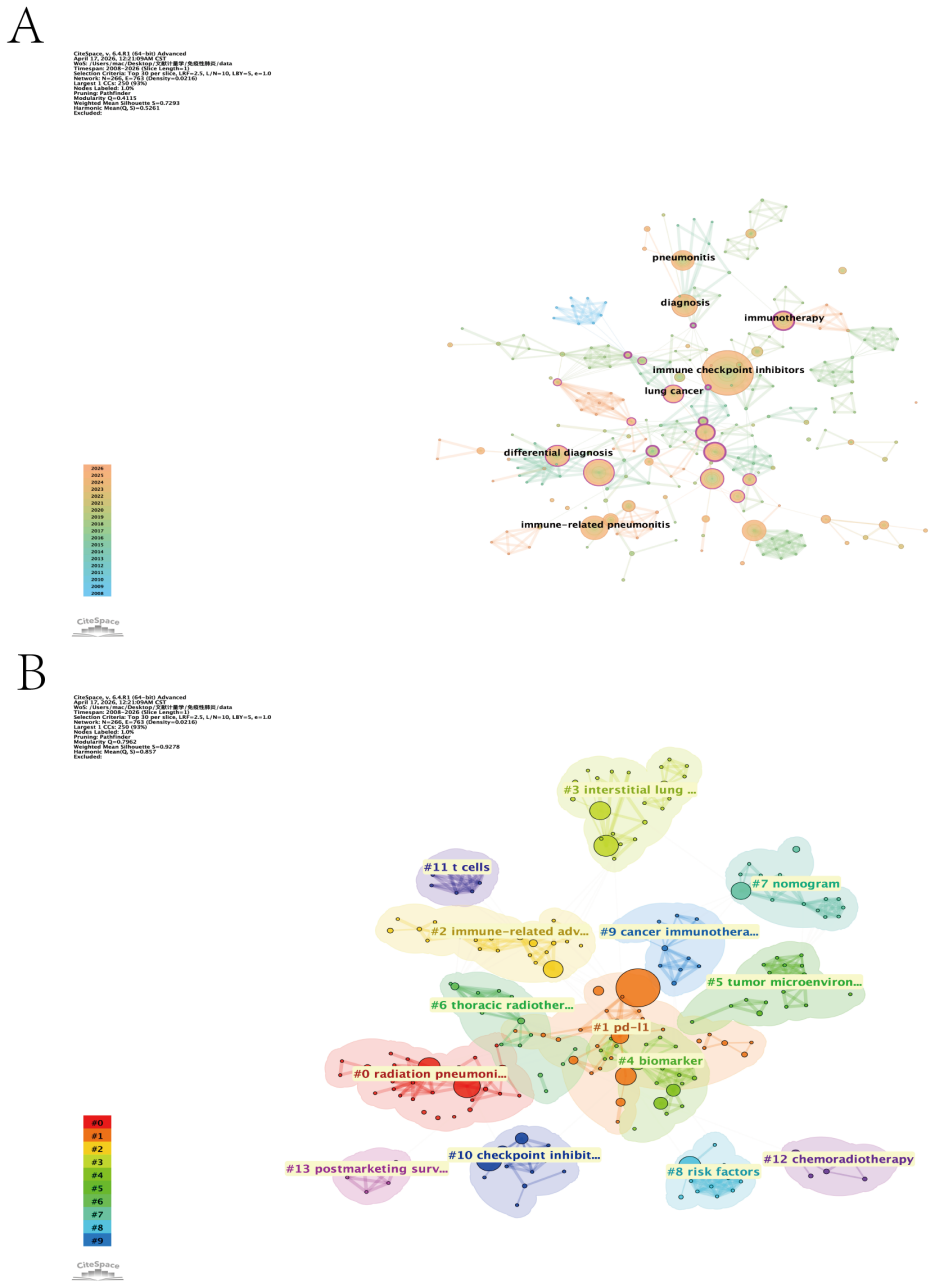
Keyword co-occurrence network and clustering structure in CIP research. **(A)** Keyword co-occurrence network. Node size reflects the frequency of keyword occurrence, and links represent co-occurrence relationships between keywords. **(B)** Keyword clustering diagram. Different colours represent different thematic clusters.

The keyword co-occurrence network reflected the main conceptual organization of CIP research. High-frequency and highly connected terms included *immune checkpoint inhibitors*, *pneumonitis*, *diagnosis*, *immunotherapy*, *lung cancer*, *differential diagnosis*, and *immune-related pneumonitis* (**Figure 7A**). These terms show that the field is centered on the diagnostic assessment of CIP in patients receiving immunotherapy, especially in the setting of lung cancer. The frequent appearance of diagnostic terms also shows that diagnostic identification and differentiation from other pulmonary disorders have become major concerns.

The clustering analysis divided the keyword network into several thematic groups (**Figure 7B**). The largest clusters included *radiation pneumonitis* (#0), *PD-L1* (#1), *immune-related adverse events* (#2), *interstitial lung disease* (#3), *biomarker* (#4), *tumor microenvironment* (#5), *thoracic radiotherapy* (#6), *nomogram* (#7), *risk factors* (#8), *cancer immunotherapy* (#9), *checkpoint inhibitor* (#10), *T cells* (#11), *chemoradiotherapy* (#12), and *postmarketing surveillance* (#13). Recent CIP studies are increasingly concerned with how pneumonitis is recognized, separated from other lung injuries, and predicted before severe disease occurs.

Several clusters are closely associated with diagnostic assessment. Radiation pneumonitis, interstitial lung disease, radiotherapy to the chest, and chemoradiotherapy reflect the difficulty of distinguishing CIP from other forms of lung injury in cancer patients. The clusters relating to biomarkers and risk factors indicate that researchers are seeking quantifiable indicators to enable diagnostic identification or prediction of disease severity. The “nomogram” cluster indicates that predictive models are being utilised to integrate clinical variables, imaging data and laboratory markers for personalized risk assessment.

The keywords cluster suggests that research into the diagnostic assessment of CIP is gradually increasing. Current studies are placing greater emphasis on imaging findings, biomarkers, treatment history, baseline lung status and predictive models, rather than relying solely on symptoms or CT findings.

### 3.8 Temporal evolution and burst keywords

**Figure 8:**
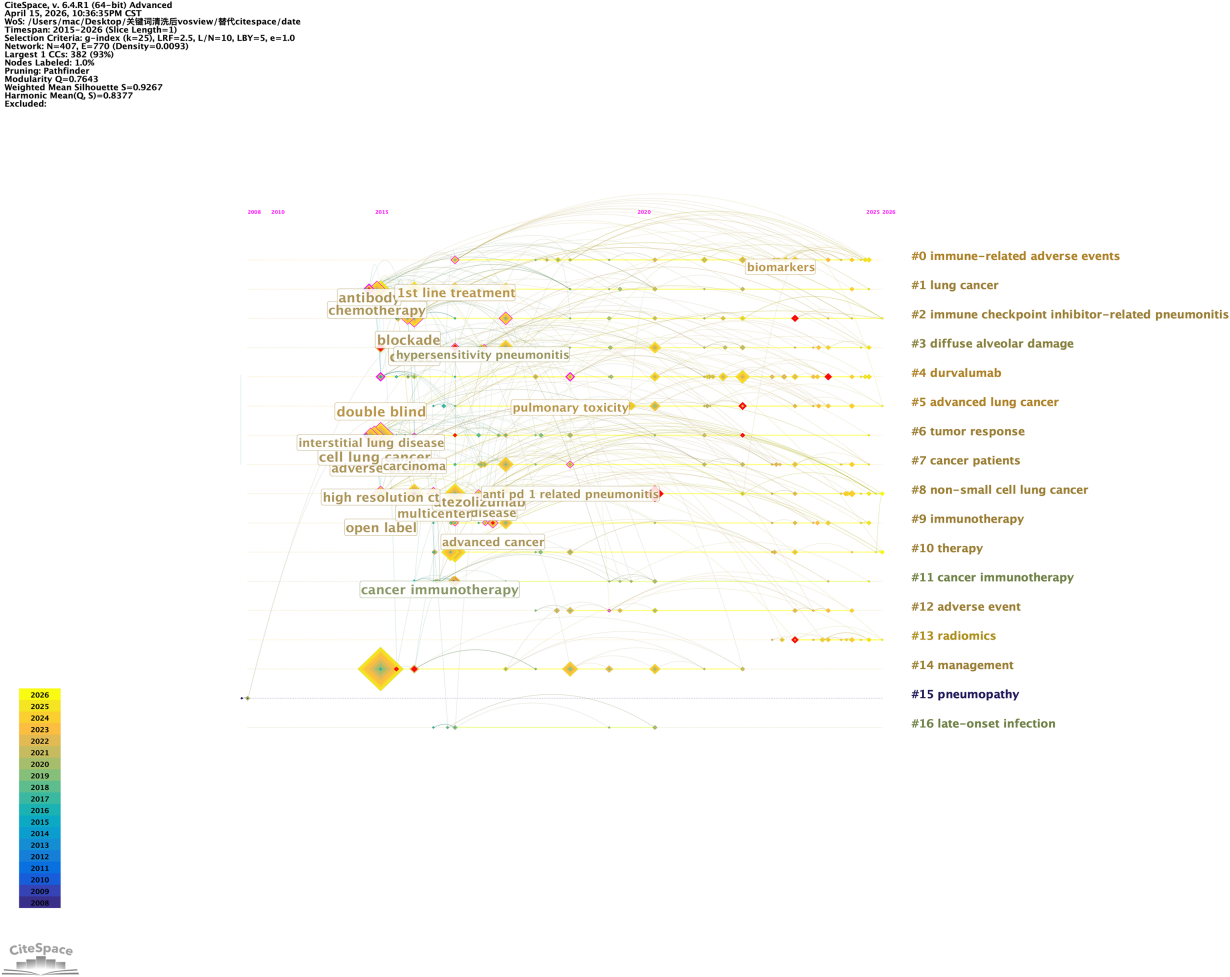
The temporal evolution of keyword clusters in CIP research. A temporal view of the main keyword clusters illustrates the temporal distribution of research themes.

A temporal view of keyword clusters reveals how research priorities have shifted over time (**Figure 8**). In the early stages, research related to CIP was closely associated with immune checkpoint inhibition in melanoma. Keywords such as T cells, metastatic melanoma, advanced melanoma, antibodies and inhibition appeared early on, reflecting the initial clinical context in which ICIs were being extensively studied and applied at that time. During this period, immune-related pneumonitis was generally discussed within the broader framework of immune-related toxicities.

As the use of ICIs in lung cancer and other solid tumors has become increasingly widespread, the focus of research has gradually shifted towards clinical identification and management. In the timeline diagram, there has been a significant increase in the frequency of terms such as “organizing pneumonia”, “management”, “lung cancer” and “differential diagnosis”. This shift reflects the growing need to distinguish CIP from infection, radiation pneumonitis, tumor progression and other treatment-related lung injuries.

Recent research areas include PD-L1 inhibitors, BAL, risk factors and predictive models. These terms have a more direct bearing on diagnostic assessment and personalized prognosis. Consequently, current research no longer focuses solely on describing clinical manifestations, but places greater emphasis on tools that can aid diagnostic assessment and risk stratification.

An analysis of burst keywords provides further evidence of this pattern (**Table 3**). Among the early burst keywords, *metastatic melanoma* had the highest trend intensity (9.33), followed by *docetaxel* (7.99), *advanced melanoma* (7.57) and *melanoma* (6.06). These bursts align with the early impact of clinical trials on melanoma and immune checkpoint inhibitors. Recently emerging burst keywords include *surviva*l and *cancer*, both of which are projected to persist from 2024 to 2026.

**Table 3:** Top 12 keywords with the strongest citation bursts in immune checkpoint inhibitor-related pneumonitis research. .

NO.	Keyword	Year	Strength	Begin	End
1	t cells	2008	3.03	2008	2019
2	metastatic melanoma	2015	9.33	2015	2020
3	antibody	2015	1.97	2015	2016
4	advanced melanoma	2016	7.57	2016	2020
5	blockade	2016	3.90	2016	2019
6	docetaxel	2016	7.99	2017	2020
7	melanoma	2017	6.06	2017	2019
8	open label	2015	5.15	2017	2019
9	organizing pneumonia	2017	4.40	2017	2020
10	management	2019	5.97	2019	2020
11	survival	2016	6.87	2024	2026
12	cancer	2016	2.33	2024	2026

Rows are arranged according to the year of burst onset (Begin year).

### 3.9 Supplementary cross-database analysis based on PubMed

The PubMed dataset was analyzed separately to provide a clinically oriented supplement to the WoSCC-based bibliometric analysis. Although the number of included PubMed records was smaller than that of the WoSCC dataset, the thematic distribution of these studies provided additional insight into how diagnostic assessment of CIP is represented within the biomedical and clinical literature.

A supplementary analysis was conducted using a manually screened non-overlapping subset of PubMed records (n = 74). After removing records overlapping with the WoSCC dataset, the remaining records were screened according to predefined inclusion and exclusion criteria (**Supplementary Table S1**). The retained articles were subsequently classified into six predefined diagnostic themes according to explicit classification criteria (**Supplementary Table S2**). The distribution of these themes is presented in **Table 4**.

**Table 4:** Clinical topic classification of the supplementary PubMed dataset.

Main topic	Number of publications	Percentage (%)
Clinical diagnosis/differential diagnosis	7	9.5%
Imaging features	27	36.5%
BAL/bronchoscopy/pathology	15	20.3%
Biomarkers/laboratory indicators	7	9.5%
Risk factors/prediction/prognosis	14	18.9%
Management/review/guideline	4	5.4%

The subject classification revealed that imaging features accounted for the largest proportion of the PubMed dataset, comprising 27 articles (36.5%). BAL/bronchoscopy/pathology was the second largest category, comprising 15 articles (20.3%), followed by risk factors/prediction/prognosis, comprising 14 articles (18.9%). Clinical diagnosis/differential diagnosis and biomarkers/laboratory markers each comprised 7 articles (9.5%), while there were 4 articles (5.4%) related to treatment/reviews/guidelines (**Table 4**; **Supplementary Table S2**). This distribution suggests that the PubMed literature places greater emphasis on practical diagnostic approaches and clinical decision-making.

Although broadly consistent with the WoSCC findings, the PubMed analysis places greater emphasis on clinically oriented diagnostic topics (**Table 5**). The WoSCC dataset outlines broader academic patterns, including collaboration networks, journal distribution, keyword clustering and temporal trends. In contrast, the PubMed dataset highlights imaging interpretation, bronchoscopy-related assessments, pathology and risk prediction. This difference is consistent with PubMed’s greater emphasis on biomedical and clinical literature.

**Table 5:** Comparison of major findings between the WoSCC and PubMed datasets.

Aspect	WoSCC	PubMed	Interpretation
Main role in this study	Primary bibliometric analysis	Supplementary clinical-topic assessment	Complementary design
Included publications	628	74	WoSCC served as the core dataset
Annual trend	Marked increase after 2019	Overall upward trend with fluctuations	Consistent temporal evolution
Main hotspots	Diagnosis, pneumonitis, differential diagnosis, and lung cancer	Imaging features, BAL/bronchoscopy/pathology, and risk prediction	PubMed was more clinically oriented
Analytical strength	Cooperation networks, journal/source analysis, and hotspot mapping	Clinically oriented thematic description	Different but complementary strengths

The findings from PubMed are generally consistent with those from WoSCC, but place greater emphasis on clinical diagnostic issues. Both sets of data indicate that diagnostic assessment, differential diagnosis and personalized risk assessment are the key focuses of recent CIP research.

## 4 Discussion

### 4.1 Overall development of CIP research

This bibliometric study reveals a marked and sustained increase in research on CIP, particularly after 2018. This trend aligns with the clinical approval and widespread use of ICIs such as Ipilimumab (28) (FDA approval in 2011), Pembrolizumab (29) and Nivolumab (30, 31) (FDA approval in 2014), and the newer ICIs approved in China such as Sintilimab (32) and Toripalimab (33), which entered clinical practice in 2018. These milestones have led to an increasing focus on the immune-related adverse effects of ICIs, particularly CIP, as more patients are treated with immunotherapy. Early studies on CIP were limited, with only one article published in 2008 (34), reflecting the limited use of ICIs at that time. However, since 2018, there has been a significant increase in the volume of relevant literature, which is consistent with the widespread use of ICIs in clinical practice and the growing attention being paid to their adverse effects. A clear increase in publication output was observed in the WoSCC dataset, whereas the PubMed dataset was used primarily to provide supplementary clinically oriented topic information.

At the same time, the increase in the volume of relevant literature since 2019 reflects a shift in understanding of CIP. Early research primarily focused on identifying pneumonitis as part of an immune-related adverse event. However, recent studies have centered on more complex clinical issues, such as how to distinguish CIP from competing pulmonary diagnoses and identify patients at risk of severe disease. Research into imaging findings (35), baseline pulmonary abnormalities (36), BAL (36), serum biomarkers (37), radiological examinations and predictive models (38) is becoming increasingly important.

At the national level, China publishes the highest number of papers, while the United States wields the greatest citation impact. This disparity highlights the distinction between publication volume and academic influence. Given that the diagnostic criteria and clinical pathways for CIP are still evolving, high-impact evidence is likely to emerge primarily from multicenter cohort studies, standardized definitions, and research applicable to diverse treatment settings. Notably, publication productivity and citation impact were not entirely consistent across countries. While China and the United States dominated in terms of publication output, countries such as the Netherlands and Ireland exhibited relatively high citation impact despite lower publication counts, highlighting the importance of research quality and influence beyond publication quantity.

With the widespread use of ICIs, particularly in combination with chemotherapy, radiotherapy and targeted therapy, our understanding of CIP continues to deepen. We are gradually recognizing that CIP is not merely an adverse event characterized by pulmonary symptoms, but rather a diagnostic issue involving multiple disciplines, including oncology, respiratory medicine, critical care medicine, radiology, pathology and immunology.

### 4.2 Research hotspots and evolving trends

The focus of CIP research has shifted significantly, moving from the mechanisms of immune checkpoint inhibition to more clinically relevant areas such as diagnostic assessment, differential diagnosis and risk prediction. Initially, CIP was closely associated with melanoma and immune checkpoint inhibition (39–42), with research primarily focusing on the underlying immune mechanisms. However, as ICI therapy has expanded to other cancers, particularly lung cancer, the focus has gradually shifted towards clinical diagnostic assessment and management (43–46).

In recent years, there has been growing international interest in biomarkers and predictive models. Biomarkers involved in inflammation and immune activation, such as KL-6, SP-D, IL-6, IL-17 and IL-10, have attracted increasing attention in studies investigating diagnostic and risk stratification of CIP (1, 22, 47–55). Furthermore, the role of imaging modalities, including PET-CT (25, 56–58) and high-resolution CT (18, 59–62), has been emphasized, as these tools are now essential for distinguishing CIP from other pulmonary conditions, such as infection, radiation pneumonitis and tumor progression.

This shift in focus from immune mechanisms to clinical diagnosis and biomarker discovery is consistent with trends in oncology. There has been a shift from general toxicity management towards personalized diagnosis and treatment based on precision medicine (62). This shift is supported by growing evidence from WoSCC and PubMed analyses, which demonstrate increasing interest in risk prediction models. Recent studies increasingly explore whether biomarkers, imaging characteristics and clinical variables can be incorporated into predictive models. However, whether such multimodal approaches can improve risk stratification requires further investigation in large clinical studies.

### 4.3 Clinical implications for diagnostic assessment

In clinical practice, it is not enough merely to identify abnormal findings in the lungs following immunotherapy. It is also essential to determine whether these abnormalities are indeed caused by CIP (63–65). This is because infections (66), radiation pneumonitis (67–70), tumor progression (71), pulmonary oedema (72), pre-existing interstitial lung disease (15) and drug-induced lung injury (73) may all produce similar clinical and radiological findings.

Chest CT remains the cornerstone of diagnostic assessment for patients with suspected CIP (7). However, its value is not limited to the mere detection of pulmonary abnormalities. Previous radiological studies, including the position paper by Johkoh et al. (10), have demonstrated that CIP presents with several characteristic imaging patterns, including organizing pneumonia (OP) (74), nonspecific interstitial pneumonia (NSIP) (75), hypersensitivity pneumonitis (HP) (76), eosinophilic pneumonia (EP) (77) and diffuse alveolar damage (DAD) (78). Among these, the OP pattern is the most common presentation, typically carrying a relatively good prognosis and responding well to corticosteroid treatment. In contrast, the NSIP and HP patterns often overlap with pre-existing interstitial lung disease, drug-induced pneumonia or infectious lesions, making differential diagnosis particularly challenging and highlighting the importance of baseline lung function status and multidisciplinary assessment. More importantly, the radiological phenotype may provide clinically relevant information beyond the scope of diagnosis. Severe manifestations, such as the DAD pattern and acute respiratory distress syndrome (ARDS)-like changes (74, 79), are associated with diffuse alveolar damage, respiratory failure, admission to the intensive care unit, and poor prognosis, and are more common in severe or fatal CIP. Therefore, radiological findings should not be regarded merely as descriptive abnormalities, but rather as phenotypic indicators that may reflect disease severity and influence subsequent treatment strategies (80). In the WoSCC and PubMed datasets, imaging-related keywords are becoming increasingly prominent, indicating that radiological assessment is shifting from a purely descriptive approach towards a phenotype-driven framework. Imaging findings are no longer merely a means of identifying pulmonary infiltrates; different imaging patterns may correspond to distinct pathophysiological mechanisms, differential diagnostic considerations and clinical courses. Against this backdrop, radiological assessment serves not only as a diagnostic tool but also as a vital component of risk stratification and prognostic evaluation (18, 80, 81). However, there is significant overlap between radiological presentations, and imaging findings alone are insufficient to establish a definitive diagnosis. Therefore, CT interpretation should always be integrated with clinical presentation, treatment history, BAL results, pathological assessment and laboratory evidence to achieve a more accurate diagnosis.

When imaging and clinical data are insufficient, bronchoscopy and BAL can provide valuable diagnostic information (82, 83). These procedures not only aid in the diagnosis of CIP, but also help to rule out infectious causes and characterize the pattern of inflammation. Previous studies have shown that bronchoalveolar lavage fluid (BALF) (84, 85) in patients with CIP is typically lymphocyte-predominant, while eosinophil and neutrophil counts, as well as the CD4/CD8 ratio (86–88), may vary between patients. More importantly, microbiological analysis of BALF helps to rule out opportunistic infections (76, 89–91), including Pneumocystis carinii infection, cytomegalovirus infection, fungal infections and mycobacterial disease, which often present with clinical features similar to CIP in immunocompromised patients. Therefore, BAL should not be regarded merely as an ancillary investigation, but rather as an integral part of the comprehensive diagnostic assessment, particularly in complex cases where imaging findings are non-specific or where differential diagnosis remains challenging.

Another issue attracting increasing attention is the role of baseline pulmonary phenotypes in determining susceptibility to CIP. Although previous studies have often grouped these conditions under the broad category of “baseline pulmonary abnormalities”, growing evidence suggests that different pulmonary phenotypes may carry varying degrees of risk. Interstitial lung abnormalities (ILA) (92), diagnosed interstitial lung disease (ILD) (93), fibrotic ILA (94) and honeycomb lung (95) collectively constitute a spectrum of interstitial abnormalities characterized by progressive impairment of lung reserve capacity and increased susceptibility to immune-mediated injury. In particular, fibrotic changes and honeycomb lung are associated with a significantly increased incidence of severe pneumonia and poorer clinical outcomes.

In addition, airway-related abnormalities should be taken into account. Chronic obstructive pulmonary disease (COPD) (96, 97) and emphysematous changes are relatively common in patients with lung cancer and have been identified as potential risk factors for CIP. In clinical practice, a history of smoking, emphysema, COPD, interstitial abnormalities and a history of radiotherapy to the chest often coexist rather than acting independently (98–101). Consequently, the incidence of CIP may reflect the accumulation of multiple pulmonary pathologies rather than the presence of any single abnormality. The analysis in this paper also suggests that clusters associated with interstitial lung disease and keywords linked to risk factors are becoming increasingly prominent, indicating that future research may move beyond single-factor associations towards phenotype-driven risk stratification and personalized monitoring strategies.

Although circulating biomarkers are useful for assessing suspected CIP and for subsequent monitoring, their limitations are also worthy of attention. Previous studies have investigated biomarkers associated with epithelial damage and immune activation, including KL-6, SP-D, IL-6, IL-10 and IL-17 (21, 86, 102–106). However, these biomarkers lack sufficient specificity to serve as independent diagnostic tools. In particular, KL-6—originally proposed as a tumor marker for lung cancer and regarded as an indicator of alveolar epithelial damage—may also show elevated levels in cases of infectious pneumonia, radiation pneumonitis, and exacerbations of pre-existing interstitial lung disease (102). Meanwhile, other inflammatory cytokines share similar limitations, as their concentrations may be influenced by a variety of inflammatory or immune-mediated conditions.

Consequently, the primary challenge in biomarker research lies not only in identifying new candidate markers, but also in enhancing diagnostic specificity and clinical applicability. Current evidence suggests that circulating biomarkers are more likely to provide complementary information when analyzed in conjunction with imaging findings, BAL results, treatment history and baseline pulmonary phenotype. Consequently, biomarker-based strategies should be regarded as part of a comprehensive diagnostic framework, rather than as standalone indicators for CIP.

Predictive models and nomograms are also useful as they provide a means of addressing multiple variables of uncertainty (38). They can assist clinicians in assessing risk prior to the development of severe disease, particularly in patients with a history of radiotherapy to the chest, pre-existing pulmonary abnormalities, those receiving combination therapy regimens, or those presenting with complex clinical presentations. The key questions are whether these models can be validated across centers and whether they can improve actual clinical decision-making, rather than merely providing statistical predictions. Most currently available nomograms and machine-learning models are primarily designed to identify patients at increased risk of developing CIP or severe CIP, rather than serving as diagnostic tools once pneumonitis is suspected (107, 108). Moreover, many of these models are based on retrospective single-center cohorts and lack external validation. Therefore, their ability to improve real-world clinical decision-making remains uncertain.

In clinical practice, the diagnosis of CIP may require the use of a variety of diagnostic tests. For example, CT scans may suggest the condition, BAL or histopathological examination can help clarify difficult cases, biomarkers can aid in monitoring, and predictive models can assist with risk stratification. Consequently, these approaches should be regarded as a comprehensive reflection of current research trends and are not yet considered diagnostic criteria. Large-scale prospective clinical studies are now required to develop comprehensive strategies that integrate imaging, BAL or histopathological assessment, biomarkers, baseline pulmonary phenotypes and predictive models, thereby providing a direction for improving clinical diagnostic capabilities.

### 4.4 Strengths and limitations

The strength of this study lies in its comprehensive bibliometric analysis, which combines data from WoSCC and PubMed. By analyzing annual publication trends, national contributions, institutional collaborations, keyword co-occurrence and temporal evolution, we provide a holistic perspective on the development of CIP research. Furthermore, the clinically oriented PubMed dataset provided additional clinically oriented insights and extended the findings derived from the WoSCC analysis, demonstrating that CIP research is increasingly aligned with clinical needs.

However, the study also has limitations. The WoSCC dataset may have excluded relevant articles indexed in other databases, and the inclusion of English-language publications only may introduce a language bias. In addition, because the final WoSCC dataset was restricted to literature with explicit diagnostic relevance to CIP, the findings should be interpreted as reflecting trends within the selected diagnostic-oriented literature rather than the entire body of CIP research. Although 628 WoSCC records were included in the analysis as the primary bibliometric dataset, certain relevant publications from other databases may not have been covered. The findings should therefore be understood as applying only to the scope of the selected databases. Furthermore, the smaller PubMed dataset was intended for supplementary thematic characterization rather than a complete bibliometric analysis, and the lack of direct merging between the two datasets may result in the loss of some details. Despite these limitations, this study provides a solid foundation for understanding the evolving landscape of CIP research.

### 4.5 Future directions

In future, the diagnosis of CIP should be based on a comprehensive assessment of various diagnostic tests and clinical manifestations. Numerous studies have analyzed CT imaging findings, serum biomarkers, BAL results, histopathological changes and clinical risk factors. Although these studies are of great value, they do not fully reflect clinical practice. Clinicians often need to synthesize multiple findings, some of which remain inconclusive, in order to determine whether CIP is the most likely diagnosis. Although studies on prognosis are receiving increasing attention in the literature on CIP, the clinical issues addressed in these studies differ from those in diagnostic assessments. The relationship between the occurrence of CIP and survival outcomes is closely linked to the complex relationship between immune-related adverse events and treatment efficacy. Furthermore, the interpretation of these associations may be influenced by methodological issues such as survivor bias and temporal bias. Consequently, studies related to prognosis should be regarded as a complement to diagnostic assessment rather than a direct extension of it. This distinction highlights the complexity of CIP research while maintaining the specific focus of diagnostic assessment.

At the same time, a degree of consensus is required regarding the standards used in various studies. For example, diagnostic terminology, descriptions of imaging modalities, biomarker thresholds, interpretation of BAL results and severity grading are not entirely consistent. This makes it difficult to pool data across multiple centers, thereby preventing larger-scale analyses.

Furthermore, patients receiving combination or sequential therapies require greater attention. In these cases, pulmonary toxicity may be associated with immunotherapy, radiotherapy, chemotherapy, targeted therapy, infection or pre-existing lung disease. Consequently, predictive models should take into account treatment sequence, radiation exposure, baseline pulmonary abnormalities, tumor type, immune status and dynamic changes during follow-up, rather than treating CIP as a single-factor event.

## 5 Conclusion

This bibliometric analysis shows that research on CIP has increased rapidly with the wider use of ICIs in oncology. The field has moved beyond the recognition of CIP as an immune-related pulmonary toxicity and is now more focused on diagnostic assessment, differential diagnosis, risk assessment, and outcome-related evaluation.

The diagnostic themes identified in this study reflect the uncertainty faced in clinical practice. Imaging findings, BAL or bronchoscopy, pathological evaluation, circulating biomarkers, baseline lung conditions, and prediction models are being studied because no single test can reliably distinguish CIP from infection, radiation pneumonitis, tumor progression, or other treatment-related lung injuries.

Overall, CIP diagnosis is becoming a multidisciplinary judgment based on integrated clinical, radiological, laboratory, and pathological information. Future progress will depend on whether these diagnostic approaches can be tested in larger clinical settings and linked to safer immunotherapy management.

## Data Availability

The original contributions presented in the study are included in the article/**Supplementary Material**. Further inquiries can be directed to the corresponding authors.
