# Supplementary material for: Diagnostic research in immune checkpoint inhibitor-related pneumonitis: a bibliometric analysis of research evolution, diagnostic focuses, and future priorities
Source: Front Oncol. 2026 Jul 13;16:1885789. doi: 10.3389/fonc.2026.1885789 (PMC13402123; doi:10.3389/fonc.2026.1885789)
Supplement: Supplementary file 2 [file Table1.docx]

Supplementary Material

Supplementary Table S1 Manual screening criteria

| **Screening domain** | **Inclusion criteria** | **Exclusion criteria** |
| --- | --- | --- |
| Disease relevance | Directly related to immune checkpoint inhibitor-related pneumonitis (CIP) | Unrelated to CIP |
| Diagnostic relevance | Diagnosis, differential diagnosis, imaging, BAL, bronchoscopy, pathology, biomarkers, laboratory markers, risk factors, predictive models | CIP mentioned only as background or as a general adverse event |
| Clinical focus | Studies addressing diagnostic assessment in the setting of ICI monotherapy or combination therapy | Pneumonitis caused exclusively by infection, radiotherapy, chemotherapy, targeted therapy, or other non-ICI drugs |
| Publication type and scope | Original articles or reviews with diagnostic relevance | Studies focusing only on treatment efficacy, prognosis, general irAEs, or management without diagnostic assessment |

Supplementary Table S2 Diagnostic theme classification criteria

| **Diagnostic theme** | **Definition** |
| --- | --- |
| Clinical diagnosis and differential diagnosis | Clinical diagnosis and differential diagnosis |
| Imaging features | CT, HRCT, PET/CT, radiomics, imaging phenotypes |
| BAL/Bronchoscopy/Pathology | BAL/Bronchoscopy/Pathology |
| Biomarkers | Serum biomarkers, cytokines, immune markers |
| Risk prediction | Risk factors, nomograms, machine learning prediction models |
| Treatment/Guidelines | Diagnostic recommendations, management guidelines |

Articles covering multiple topics are categorised according to their primary methodology

Supplementary Table S3 Discrepancy-resolution results for the WoSCC screening process

| **Dataset** | **Records screened** | **Discordant records** | **Resolved by discussion** | **Adjudicated by third reviewer** | **Final included records** | **Final excluded records** |
| --- | --- | --- | --- | --- | --- | --- |
| WoSCC | 887 | 45 | 32 | 13 | 628 | 259 |
| PubMed | 367 | 34 | 22 | 12 | 74 | 293 |

Supplementary Table S4 Inter-rater reliability of thematic classification

| **Measure** | **Value** |
| --- | --- |
| Number of articles classified | 74 |
| Cohen's κ coefficient | 0.862 |
| Standard error | 0.046 |
| Approximate T value | 14.902 |
| P value | <0.001 |
