# Supplementary material for: Diagnostic research in immune checkpoint inhibitor-related pneumonitis: a bibliometric analysis of research evolution, diagnostic focuses, and future priorities
Source: Front Oncol. 2026 Jul 13;16:1885789. doi: 10.3389/fonc.2026.1885789 (PMC13402123; doi:10.3389/fonc.2026.1885789)
Supplement: Supplementary file 3 [file Table2.docx]

Supplementary Material

Web of Science:

Searches:

1:TS = (("immune checkpoint inhibitor*" OR "checkpoint inhibitor*" OR ICI* OR

"PD-1" OR "PD-L1" OR "CTLA-4" OR pembrolizumab OR nivolumab OR atezolizumab OR durvalumab OR ipilimumab)AND(pneumonitis OR"immune-related pneumonitis" OR"checkpoint inhibitor pneumonitis" OR"ICI-related pneumonitis" OR"immunotherapy-related pneumonitis")AND(diagnos* OR "early diagnos*" OR detect* OR recognit* ORbiomarker* OR imaging OR radiology OR CT OR"bronchoalveolar lavage" OR BALF OR"peripheral blood" OR "blood marker*" OR "risk factor*" OR "prediction"))

Time Span: From the establishment of the database to 17 March 2026

Export Format: Excel format and Plain text file

Record Saving: Full records

Search in: Web of Science Core Collection

Collections: Web of Science Core Collection

Date Run: Tuesday, 17 March 2026

Results: 982

Select “Article” and 'Review'

Language Filter: English

Results: 887

PubMed:

("Immune Checkpoint Inhibitors"[Mesh] OR "immune checkpoint inhibitor*"[tiab] OR "checkpoint inhibitor*"[tiab] OR ICI[tiab] OR ICIs[tiab] OR PD-1[tiab] OR PD-L1[tiab] OR CTLA-4[tiab] OR pembrolizumab[tiab] OR nivolumab[tiab] OR atezolizumab[tiab] OR durvalumab[tiab] OR ipilimumab[tiab])AND( "Pneumonitis"[Mesh] OR pneumonitis[tiab] OR "checkpoint inhibitor pneumonitis"[tiab] OR "immune checkpoint inhibitor-related pneumonitis"[tiab] OR "immune-related pneumonitis"[tiab])AND( "early diagnos*"[tiab] OR diagnos*[tiab] OR "differential diagnos*"[tiab] OR biomarker*[tiab] OR imaging[tiab] OR radiolog*[tiab] OR "computed tomography"[tiab] OR "bronchoalveolar lavage"[tiab] OR BALF[tiab] OR "risk factor*"[tiab] OR prediction[tiab])AND English[lang]

Date Run: Thursday, 16 April 2026

Results: 812
