# Supplementary material for: Diagnostic research in immune checkpoint inhibitor-related pneumonitis: a bibliometric analysis of research evolution, diagnostic focuses, and future priorities
Source: Front Oncol. 2026 Jul 13;16:1885789. doi: 10.3389/fonc.2026.1885789 (PMC13402123; doi:10.3389/fonc.2026.1885789)
Supplement: Supplementary file 8 [file Table7.docx]

Supplementary Material

# Edit this file in Excel or Notepad++ and save it as citespace.alias

# Modify the first column as the label to retain

# Remove lines that don't need changes, or simply leave them alone

# Entries are arranged from the most cited to the least cited. You may only need to modify the first few lines

immune checkpoint inhibitors nivolumab

adverse events adverse events

immune checkpoint inhibitors immune checkpoint inhibitors

pneumonitis pneumonitis

immune checkpoint inhibitors pembrolizumab

docetaxel docetaxel

lung cancer lung cancer

immune checkpoint inhibitors immune checkpoint inhibitor

immunotherapy immunotherapy

non-small cell lung cancer cell lung cancer

Therapy therapy

immune checkpoint inhibitors ipilimumab

risk factors risk factors

immune-related adverse events immune-related adverse events

cancer cancer

chemotherapy chemotherapy

adverse events toxicity

non-small cell lung cancer non-small cell lung cancer

immune checkpoint inhibitors death 1

open label open label

management management

immune-related pneumonitis inhibitor related pneumonitis

adverse events safety

differential diagnosis interstitial lung disease

blockade blockade

differential diagnosis radiation pneumonitis

adverse events efficacy

disease disease

survival survival

radiotherapy radiotherapy

association association

immune-related pneumonitis checkpoint inhibitor pneumonitis

multicenter multicenter

immune checkpoint inhibitors checkpoint inhibitors

immune-related adverse events immune-related adverse event

melanoma melanoma

metastatic melanoma metastatic melanoma

risk factors risk

advanced cancer advanced cancer

advanced melanoma advanced melanoma

diagnosis diagnosis

chemoradiotherapy chemoradiotherapy

antibody antibody

immune checkpoint inhibitors anti pd 1

chemoradiation chemoradiation

immune checkpoint inhibitors atezolizumab

organizing pneumonia organizing pneumonia

immune checkpoint inhibitors pd 1

case report case report

radiation therapy radiation therapy

pulmonary fibrosis pulmonary fibrosis

outcome outcm

cancer patients cancer patients

idiopathic pulmonary fibrosis idiopathic pulmonary fibrosis

adverse events pulmonary toxicity

immune-related pneumonitis checkpoint inhibitor-related pneumonitis

non-small cell lung cancer non-small-cell lung cancer

concurrent chemoradiotherapy concurrent chemoradiotherapy

non-small cell lung cancer nsclc

diagnosis computed tomography

immune-related pneumonitis immune checkpoint inhibitor-related pneumonitis

injury injury

diagnosis features

diagnosis ct

fibrosis fibrosis

inhibitors inhibitors

immune checkpoint inhibitors durvalumab

immune checkpoint inhibitors combined nivolumab

t cells t cells

combination combination

immune checkpoint inhibitors anti ctla 4

immune immune

immune checkpoint inhibitors programmed death 1

immune checkpoint inhibitors checkpoint inhibitor

1st line treatment 1st line treatment

impact impact

thoracic radiotherapy thoracic radiotherapy

immune-related adverse events immune-related adverse events (iraes)

hypersensitivity pneumonitis hypersensitivity pneumonitis

cancer immunotherapy cancer immunotherapy

immune-related pneumonitis anti pd 1 related pneumonitis

expression expression

patient patient

double blind double blind

adverse events lung toxicity

diagnosis high resolution ct

concurrent concurrent

interstitial pneumonia interstitial pneumonia

society society

diagnosis criteria

tumor response tumor response

diagnosis guidelines

body radiation therapy body radiation therapy

diagnosis prediction

immune checkpoint inhibitors pd-1 inhibitor

radiation recall pneumonitis radiation recall pneumonitis

diagnosis radiomics

risk factors risk factor

phase 3 phase 3

immune related adverse events immune related adverse events

clinical outcome clinical outcm

metaanalysis metaanalysis

recurrent recurrent

diagnosis radiographic patterns

immune checkpoint inhibitors anti pd 1 antibody

diagnosis machine learning

diagnosis bronchoalveolar lavage

diagnosis classification

consolidation consolidation

acute exacerbation acute exacerbation

immune-related pneumonitis checkpoint inhibitor-related pneumonitis (cip)

phase ii phase ii

immune-related pneumonitis immune-related pneumonitis

diagnosis inflammation

kl 6 kl 6

interstitial lung abnormalities interstitial lung abnormalities

interstitial pneumonitis interstitial pneumonitis

japanese patients japanese patients

drug related pneumonitis drug related pneumonitis

adverse events long term safety

breast cancer breast cancer

diagnosis bronchoalveolar lavage fluid

myocarditis myocarditis

immune checkpoint inhibitors checkpoint blockade

immune-related pneumonitis checkpoint inhibitor-associated pneumonitis

immune checkpoint inhibitors death ligand 1

differential diagnosis infection

lung lung

diagnosis model

immune checkpoint inhibitors immune checkpoint inhibitor (ici)

immune checkpoint inhibitors pd-1 inhibitors

immune-related pneumonitis drug-induced pneumonitis

mechanisms mechanisms

carcinoma carcinoma

real world data real world data

events events

immune checkpoint inhibitors ipilimumab therapy

adverse effects adverse effects

stage iii stage iii

response criteria response criteria

immune checkpoint inhibitors pd 1/pd l1 inhibitors

exacerbation exacerbation

solid tumors solid tumors

radiation recall radiation recall

advanced melanoma patients advanced melanoma patients

acute myeloid leukemia acute myeloid leukemia

adverse drug reactions adverse drug reactions

death death

pulmonary function pulmonary function

activation activation

camrelizumab camrelizumab

tyrosine kinase inhibitor tyrosine kinase inhibitor

bronchiolitis obliterans bronchiolitis obliterans

deep learning deep learning

american thoracic society american thoracic society

stage stage

diffuse alveolar damage diffuse alveolar damage

autoimmune autoimmune

b7 h1 b7 h1

carboplatin carboplatin

cisplatin cisplatin

checkpoint checkpoint

adverse event adverse event

antibody treatment antibody treatment

mammalian target mammalian target

immune checkpoint inhibitors immune checkpoint inhibitors (icis)

adverse effect adverse effect

regulatory t cells regulatory t cells

high dose radiation high dose radiation

asthma asthma

immune-related adverse events immune-related adverse events (irae)

monotherapy monotherapy

disproportionality analysis disproportionality analysis

phase iii trial phase iii trial

non-small-cell lung non-small-cell lung

immune checkpoint inhibitors anti pd 1 therapy

diagnosis computed tomography (ct)

cells cells

immune checkpoint inhibitors pd l1 expression

recurrent pneumonitis recurrent pneumonitis

immune-related adverse effects immune-related adverse effects

induced pulmonary disease induced pulmonary disease

colitis colitis

respiratory distress syndrome respiratory distress syndrome

gastric cancer gastric cancer

malignant melanoma malignant melanoma

immune checkpoint inhibitors pd 1 blockade

to lymphocyte ratio to lymphocyte ratio

immune checkpoint inhibitors durvalumab consolidation

autoimmune hypophysitis autoimmune hypophysitis

tyrosine kinase inhibitors tyrosine kinase inhibitors

non-small cell lung cancer stage iii nsclc

systemic sclerosis systemic sclerosis

sex sex

population population

abnormality abnormality

autoantibody autoantibody

immune-related pneumonitis checkpoint inhibitor pneumonitis (cip)

antitumor immunity antitumor immunity

immune checkpoint inhibitors anti-pd-1 antibody

advanced lung cancer advanced lung cancer

immune checkpoint inhibitors immune checkpoint blockade

cell cell

rheumatoid arthritis rheumatoid arthritis

immune-related pneumonitis immune checkpoint inhibitor pneumonitis

interleukin 6 interleukin 6

differential diagnosis radiation pneumonitis (rp)

pathogenesis pathogenesis

diagnosis biomarkers

artificial intelligence artificial intelligence

immune checkpoint inhibitors pd 1 checkpoint expression

immune checkpoint inhibitors programmed cell death 1

emphysema emphysema

oncology oncology

statement statement

thoracic radiation thoracic radiation

acute kidney injury acute kidney injury

immune related pneumonitis immune related pneumonitis

tgf beta 1 gene tgf beta 1 gene

immune checkpoint inhibitors nivolumab plus ipilimumab

immune checkpoint inhibition immune checkpoint inhibition

radiation radiation

clinical features clinical features

t cell activation t cell activation

diseases diseases

differential diagnosis progression

lung neoplasms lung neoplasms

placebo placebo

predictors predictors

immune related response immune related response

differential diagnosis interstitial lung diseases

differential diagnosis drug-induced interstitial lung disease

receptor receptor

postmarketing surveillance postmarketing surveillance

diagnosis biomarker

th17 cells th17 cells

apoptosis apoptosis

immune checkpoint inhibitors ctla 4 blockade

autoimmunity autoimmunity

molecular targeted therapy molecular targeted therapy

differential diagnosis differential diagnosis

immune checkpoint inhibitors anti-pd-1 monoclonal antibodies

albumin albumin

diagnostic value diagnostic value

immune checkpoint inhibitors anti-pd-1 therapy

autoimmune disorders autoimmune disorders

antiangiogenic therapy antiangiogenic therapy

antifibrotic treatment antifibrotic treatment

a case-control study a case-control study

axis axis

aberrant pathway activation aberrant pathway activation

allograft recipients allograft recipients

braf braf

antibody drug conjugate antibody dug conjugate

brentuximab vedotin brentuximab vedotin

antineoplastic agents antineoplastic agents

epidermal growth factor receptor epidermal growth factor receptor

acetylcholine receptors acetylcholine receptors

disseminated vzv disseminated vzv

monoclonal antibodies monoclonal antibodies

antineoplastic agent antineoplastic agent

renal disease renal disease

induced hypophysitis induced hypophysitis

class ii class ii

b cell malignancy b cell malignancy

case-control study case-control study

immune checkpoint inhibitors death 1 blockade

guideline version 1.1 guideline version 1.1

immune checkpoint inhibitors pd-1/pd-l1 checkpoint

auto-reactive antibodies auto-reactive antibodies

immune checkpoint inhibitors cell death 1

adverse events report adverse events report

anti-programmed death-1 therapy anti-programmed death-1 therapy

distinct roles distinct roles

anti-thyroid autoantibodies anti-thyroid autoantibodies

adults adults

differential diagnosis bacterial infections

conventional microbiological testing conventional microbiological testing

mrl fas(lpr) mice mrl fas(lpr) mice

absolute eosinophil count (aec) absolute eosinophil count (aec)

anti-angiogenic therapy anti-angiogenic therapy

1 antibody 1 antibody

immune checkpoint inhibitors anti-pd-1 blockade

check-point inhibitor check-point inhibitor

immune checkpoint inhibitors anti ctla 4 antibody

16s rrna 16s rrna

avoidance avoidance

adverse events amiodarone pulmonary toxicity

aberrant basaloid cell aberrant basaloid cell

acute respiratory distress syndrome acute respiratory distress syndrome

malignant pleural mesothelioma malignant pleural mesothelioma

american college american college

adverse drug event adverse drug event

immune checkpoint inhibitors anti-pdl-1 monoclonal antibodies

dabrafenib plus trametinib dabrafenib plus trametinib

clinically amyopathic dermatomyositis clinically amyopathic dermatomyositis

area area

diagnosis balf (bronchoalveolar lavage fluid)

non-small cell lung cancer advanced nsclc

bronchiectasis bronchiectasis

immune-related side-effect immune-related side-effect

complete remission complete remission

murine lupus murine lupus

copper 64 copper 64

clinical practice clinical practice

positron emission tomography positron emission tomography

aspiration pneumonia aspiration pneumonia

complications complications

active antiretroviral therapy active antiretroviral therapy

complication probability complication probability

checkpoint protein checkpoint protein

elastase inhibitor elastase inhibitor

differential diagnosis bacterial infection

lpr mice lpr mice

bevacizumab bevacizumab

autoimmune thyroiditis autoimmune thyroiditis

immune checkpoint inhibitors anti-ctla-4 antibodies

central airway tumor invasion central airway tumor invasion

immune checkpoint inhibitors anti pd-1/pd-l1

diagnosis body ct

camel camel

anti-cytotoxic t-lymphocyte-associated protein-4 therapy anti-cytotoxic t-lymphocyte-associated protein-4 therapy

autoimmune disease autoimmune disease

minimal change disease minimal change disease

active ulcerative colitis active ulcerative colitis

mammalian target of rapamycin mammalian target of rapamycin

b lymphocytes b lymphocytes

antibody-drug conjugate antibody-drug conjugate

adverse drug events adverse drug events

check point inhibitors check point inhibitors

b7 family b7 family

avelumab avelumab

growth factor growth factor

chemokine chemokine

appearance appearance

advanced lung cancer advanced lung cancer&nbsp

camp camp

bleomycin induced pneumonitis bleomycin induced pneumonitis

dimerization inhibitor dimerization inhibitor

autoimmune antibody autoimmune antibody

cancer therapy cancer therapy

cns demyelination cns demyelination

acquired immunodeficiency syndrome acquired immunodeficiency syndrome

immune checkpoint inhibitors anti pd-1 antibody

immune checkpoint inhibitors anti-pd-1/pd-l1 therapy

antibody imaging antibody imaging

administrative claims data administrative claims data

car t cells car t cells

non-invasive imaging non-invasive imaging

clinical characteristics clinical characteristics

anti-tumor therapy anti-tumor therapy

case reports case reports

antitumor activity antitumor activity

anemia anemia

immune checkpoint inhibitors pd-1 antibody

acyclovir acyclovir

alveolar hemorrhage alveolar hemorrhage

cohort cohort

legionella pneumonia <italic>legionella pneumonia</italic>

anti-programmed death-1 anti-programmed death-1

binding binding

bispecific t-cell engager bispecific t-cell engager

immune checkpoint inhibitors antibody (anti-pd-1 antibody)

acquired resistance acquired resistance

dilated cardiomyopathy dilated cardiomyopathy

antibody drug conjugate antibody drug conjugate

airway obstruction airway obstruction

airway airway

blood counts bllod counts

acute lung injury acute lung injury

b cells b cells

computational pathology computational pathology

cardiac troponin i cardiac troponin i

anti-tumour effect anti-tumour effect

amifostine amifostine

acute management acute management

anaplastic lymphoma kinase-inhibitors anaplastic lymphoma kinase-inhibitors

alter alter

acute pneumonitis acute pneumonitis

checkpoint immune therapy checkpoint immune therapy

apoptotic cells apoptotic cells

antiplatelet drug antiplatelet drug

b cell b cell

gene gene

colony stimulating factor colony stimulating factor

atrioventricular block atrioventricular block
